# Migratory herds of wildebeests and zebras indirectly affect calf survival of giraffes

**DOI:** 10.1002/ece3.2561

**Published:** 2016-10-25

**Authors:** Derek E. Lee, Bernard M. Kissui, Yustina A. Kiwango, Monica L. Bond

**Affiliations:** ^1^ Wild Nature InstituteHanoverNHUSA; ^2^ Tarangire Lion ProjectSchool for Field StudiesKaratuTanzania; ^3^ Tanzania National ParksArushaTanzania

**Keywords:** asynchronous reproduction, juvenile survival, life history, mammal, match–mismatch, natural enemy, population dynamics, predator–prey ratio, prey switching

## Abstract

1. In long‐distance migratory systems, local fluctuations in the predator–prey ratio can exhibit extreme variability within a single year depending upon the seasonal location of migratory species. Such systems offer an opportunity to empirically investigate cyclic population density effects on short‐term food web interactions by taking advantage of the large seasonal shifts in migratory prey biomass.
2. We utilized a large‐mammal predator–prey savanna food web to evaluate support for hypotheses relating to the indirect effects of “apparent competition” and “apparent mutualism” from migratory ungulate herds on survival of resident megaherbivore calves, mediated by their shared predator. African lions (*Panthera leo*) are generalist predators whose primary, preferred prey are wildebeests (*Connochaetes taurinus*) and zebras (*Equus quagga*), while lion predation on secondary prey such as giraffes (*Giraffa camelopardalis*) may change according to the relative abundance of the primary prey species.
3. We used demographic data from five subpopulations of giraffes in the Tarangire Ecosystem of Tanzania, East Africa, to test hypotheses relating to direct predation and indirect effects of large migratory herds on calf survival of a resident megaherbivore. We examined neonatal survival via apparent reproduction of 860 adult females, and calf survival of 449 giraffe calves, during three precipitation seasons over 3 years, seeking evidence of some effect on neonate and calf survival as a consequence of the movements of large herds of migratory ungulates.
4. We found that local lion predation pressure (lion density divided by primary prey density) was significantly negatively correlated with giraffe neonatal and calf survival probabilities. This supports the apparent mutualism hypothesis that the presence of migratory ungulates reduces lion predation on giraffe calves.
5. Natural predation had a significant effect on giraffe calf and neonate survival, and could significantly affect giraffe population dynamics. If wildebeest and zebra populations in this ecosystem continue to decline as a result of increasingly disrupted migrations and poaching, then giraffe calves will face increased predation pressure as the predator–prey ratio increases. Our results suggest that the widespread population declines observed in many migratory systems are likely to trigger demographic impacts in other species due to indirect effects like those shown here.

## 1 Introduction

The role of indirect trophic interactions in the ecology and evolution of organisms is a long‐standing topic among ecologists (Estes, Brashares, & Power, 2013; Schmitz, Hambäck, & Beckerman, 2000). Indirect interactions arise when the effect of one species on another is mediated by the action of a third species (Abrams, 1987; Wootton, 1994). Shared predation in two or more prey species is an indirect interaction often discussed in natural communities (Holt, 1977; Holt & Lawton, 1994; Paine, 1966). Theoretically, increases in the density of one prey species can decrease the predator's functional response to a second prey species, either due to satiation or prey switching (Murdoch, 1969; Murdoch & Oaten, 1975), resulting in a one‐way indirect interaction called “apparent mutualism” (Abrams, 1987; Abrams & Matsuda, 1996). Alternatively, increases in density of one prey can lead to locally increased predator numbers or feeding rate, resulting in increased predation on a second prey species (Abrams, 1995; Holt, 1977; Holt & Kotler, 1987; Holt & Lawton, 1994), a one‐way indirect effect called “apparent competition” because patterns generated by this process can appear to be the result of competition.

Whereas there is currently more empirical evidence for apparent competition (Chaneton & Bonsall, 2000), empirical findings supporting apparent mutualism are fewer and none involve large mammals (e.g., Carslake, Cornulier, Inchausti, & Bretagnolle, 2011; Sundararaj, McLaren, Morris, & Goyal, 2012; Tack, Gripenberg, & Roslin, 2011). Theoretical studies have predicted apparent mutualism (Abrams & Matsuda, 1996; Joly & Patterson, 2003), particularly when population density of one prey species exhibits cyclic population fluctuations (Abrams, Holt, & Roth, 1998; Barraquand, New, Redpath, & Matthiopoulos, 2015).

The transfer of energy via long‐distance migration of primary prey species can modify interactions between predators and secondary prey seasonally, and thus affect the functioning of food webs (Giroux et al., 2012; Loreau & Holt, 2004). In long‐distance migratory systems, local fluctuations in the predator–prey ratio within one portion of the migratory range can exhibit extreme variability within a single year depending upon the seasonal location of the migratory species (Bolger, Newmark, Morrison, & Doak, 2008; Fryxell, Greever, & Sinclair, 1988). These systems offer an opportunity to empirically investigate cyclic population density effects on short‐term food web interactions by taking advantage of the large seasonal shifts in migratory prey species biomass.

We used a large‐mammal predator–prey food web in the savanna Tarangire Ecosystem (TE) of northern Tanzania, East Africa, to examine hypotheses relating to the direct effect of predation and the indirect effect of migratory ungulate herds on population dynamics of a resident megaherbivore. African lions (*Panthera leo*) are generalist predators whose primary, preferred prey are wildebeests (*Connochaetes taurinus*) and zebras (*Equus quagga*), while lion predation on secondary prey such as giraffes (*Giraffa camelopardalis*) may change according to the relative abundance and vulnerability of the primary prey species (Hayward & Kerley, 2005; Hopcraft, Sinclair, & Packer, 2005; Owen‐Smith & Mills, 2008). While adult giraffes are not the preferred prey of lions in this area (B. Kissui, unpublished data), lions are the most important natural predators of giraffes, and may be the primary cause of death for giraffe calves (Dagg, 2014; Dagg & Foster, 1976; Strauss & Packer, 2013).

Our TE study area encompassed five distinct but connected sites surveyed in three seasons each year yielding spatiotemporally contrasting levels of lion density and primary prey density, providing an opportunity to examine how variation in predator and migratory primary prey densities affected neonatal and calf survival in a secondary prey species, giraffes. Our study sites experience large seasonal variation in ungulate density and biomass as migratory herds of thousands of wildebeests and zebras move between separate wet‐ and dry‐season ranges (Lamprey, 1964; Morrison & Bolger, 2014), leading to spatiotemporal variation in predation pressure among local giraffe subpopulations. Our objective was to examine whether and how changes in predation pressure that result from the presence or absence of migratory herds might affect giraffe demographic traits of neonatal survival and calf survival.

Variation in survival of juveniles plays an important role in population dynamics of ungulates (Coulson, Albon, Guinness, Pemberton, & Clutton‐Brock, 1997; Gaillard, Festa‐Bianchet, Yoccoz, Loison, & Toigo, 2000). Juvenile survival is more sensitive to environmental variation than adult survival in some populations of large herbivores (Eberhardt, 2002; Gaillard et al., 2000), and often explains a large part of the variance in parents’ fitness, as measured by lifetime reproductive success (Clutton‐Brock, Albon, & Guinness,^1988^).

We used individual‐based giraffe data collected as encounter histories constructed from seasonal photographic capture–mark–recapture (PCMR) data for wild Masai giraffes (*G. c. tippelskirchii*) to directly estimate calf survival during the 4‐month interval after first detection in every site–season combination. We also estimated neonatal survival for the period between birth and our seasonal surveys via variation in apparent reproduction. We ranked three spatiotemporal covariate models of local lion density, local primary lion prey density (migratory ungulates: wildebeests and zebras), and local lion predation pressure (the ratio of lions to migratory ungulates), to determine whether they explained site‐ and season‐specific giraffe demographic traits of neonatal survival and calf survival. The three models represented three possible relationships for how giraffe subpopulations may be directly or indirectly influenced by lion predation and migratory ungulates. We predicted: (1) local lion density is negatively correlated with giraffe demographic traits indicating a direct predation effect. (2) Giraffe demographic traits are correlated with local migratory ungulate density such that a positive correlation would indicate apparent mutualism and a negative correlation would signal apparent competition. (3) Local lion predation pressure is correlated with giraffe demographic traits such that a negative correlation supports apparent mutualism and a positive correlation supports apparent competition.

## 2 Materials and Methods

### 2.1 Study system

Giraffes are large (830–1,000 kg), long‐lived, nonmigratory, nonterritorial, browsing ruminants, that give birth aseasonally in any month (Dagg, 2014; Dagg & Foster, 1976). Their main natural predators are African lions and spotted hyenas (*Crocuta crocuta*: Dagg & Foster, 1976; Dagg, 2014). Female giraffes attain sexual maturity at ~5 years of age and may breed up to age 20 (Dagg, 2014; Dagg & Foster, 1976), with an observed birth interval of 620 ± 49 days (Bercovich & Berry, 2009; del Castillo, Bashaw, Patton, Rieches, & Bercovitch, 2005). Young typically are weaned at 9 months of age and are independent at 14 months (Langman, 1977).

The TE is a tropical savanna‐woodland ecosystem (Lamprey, 1964) and supports the second highest density of giraffes in Tanzania (Stoner et al., 2007). The TE is in the eastern branch of the Great Rift Valley and encompasses roughly 30,000 km^2^ (Borner, 1985; Prins, 1987). The TE has three seasons, short rains (November–February), long rains (March–June), and dry (July–October), with mean total annual rainfall of 650 mm (1980–2009), range = 312–1398 mm (Foley & Faust, 2010).

**Figure 1:**
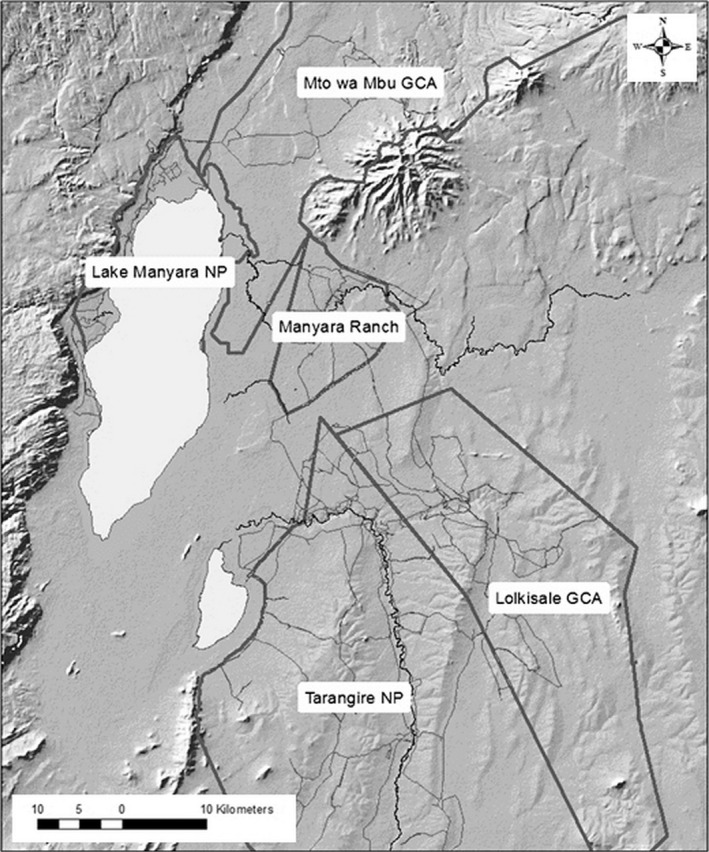
Study area in the Tarangire Ecosystem of northern Tanzania. Thick gray lines delineate the five sites sampled, thin gray lines are roads and tracks, black lines are rivers and watercourses, and light gray areas are lakes

Our study area in the TE included five sites (Figure 1): Tarangire National Park (TNP), Lake Manyara National Park (LMNP), Manyara Ranch Conservancy (MRC), Lolkisale Game Controlled Area (LGCA), and Mto wa mbu Game Controlled Area (MGCA). The two national parks had stricter enforcement of antipoaching laws and no permanent settlements, MRC was a private ranch/wildlife conservancy with some antipoaching activity and a moderate density of pastoralists and livestock but no permanent settlements, and the two Game Controlled Areas had few antipoaching activities, high density of pastoralists and livestock, agriculture and permanent human settlements, and wildlife harvesting via subsistence and trophy hunting, although hunting of giraffes was legally prohibited (Nelson et al., 2010).

The sites differed spatially in lion density and spatiotemporally in primary prey biomass as migratory herds congregated in TNP during the dry season and dispersed to breeding grounds mostly outside our study area during the wet seasons. Three sites (NPs and MRC) had higher lion densities due to active predator protection policies. Trophy hunting of lions and pastoralist activities in the two GCA sites resulted in lower lion densities in these sites (Davidson, Valeix, Loveridge, Madzikanda, & Macdonald, 2011). Large herds of primary prey species (~20,000 wildebeests and zebras) migrate seasonally into TNP in the dry season, and out to the GCAs during the short and long rains. This migration attracts some predators to follow the migratory herds, but most remain resident (B. Kissui, unpublished). Some migratory wildebeests and zebras move out of LMNP during the short and long rains, but unlike TNP, this park retains some resident primary prey during the dry season. MRC maintains a relatively constant primary prey and predator population. Other large ungulate species that inhabit the TE such as buffalo (*Syncerus caffer*) and eland (*Taurotragus oryx*) are also preferred lion prey (Hayward & Kerley, 2005; Hopcraft et al., 2005; Owen‐Smith & Mills, 2008), but buffalo and eland densities varied little across the site–seasons we examined and both buffalo and eland densities were substantially lower overall compared with wildebeest and zebra densities (D. Lee, unpublished), so they were not included in this study.

### 2.2 Spatiotemporal covariate models

To examine the evidence for lion predation and indirect effects of migratory herds on giraffe neonatal survival and calf survival, we ranked three spatiotemporal covariate models: lion density, primary prey density, and lion predation pressure (lion density/primary prey density), in addition to a constant null model. Because our survey designs for lions and primary prey are not as random and replicated as suggested for achieving maximum accuracy and precision (Otis, Burnham, White, & Anderson, 1978; Buckland Anderson, Burnham, and Laake 2005), we only claim that our site‐ and season‐specific density index values reflect the variation in relative densities rather than absolute densities. Thus, our covariate models reflect the season‐ and site‐specific variation in lion density, primary prey density, and predation pressure (Table 1).

**Table 1:** Spatiotemporal covariates of lion density, primary prey density (migratory herds), and lion predation pressure (lion density/primary prey density) in five sites and across three seasons within the Tarangire Ecosystem, Tanzania 2012–2014

	LMNP	MRC	TNP	LGCA	MGCA
*Lion density*					
Dry	20.5	14	9.3	1.4	1.4
Short rains	20.5	14	8.3	1.4	1.4
Long rains	20.5	14	8.3	2.4	2.4
*Primary prey density*					
Dry	10	15	100	0	0
Short rains	5	15	0	10	10
Long rains	5	15	0	15	15
*Predation pressure*					
Dry	2	1	0.1	1	1
Short rains	4	1	8	0.1	0.1
Long rains	4	1	8	0.2	0.2

Lion densities are number per 100 km^2^, prey densities are number per km^2^.

We estimated lion densities (number of individuals per 100 km^2^) for each season–site combination from lion monitoring data collected in TNP, LGCA, and MRC during 2010–2013. All lion prides in northern TNP and adjacent areas of LGCA have been identified and monitored regularly since 2003 (Packer et al., 2011). B. Kissui attempted to locate all known lion prides and individuals in TNP, LGCA, and MRC every 2 weeks using regular surveys, radio collars, GPS collars, and ancillary information from national parks staff, tourism operators, and local informants. Staff ecologists from LMNP monitored lion prides during monthly surveys of all large mammals with ancillary information from national park staff, tourism operators, and local informants. Lion density in MGCA was estimated from irregular surveys of the area by professional hunting operators augmented with estimates from LGCA with similar habitat and land‐use management. No error estimate was available for lion densities as only a single estimator of density was calculated for each season–site combination.

We computed seasonal site‐specific densities of primary lion prey (wildebeests and zebras) from road transect distance‐sampling surveys we performed during giraffe surveys between January 2012 and October 2014. We analyzed data from each site and species independently, but we combined the density (number of individuals per km^2^) of all primary prey species and used this as the covariate value in each season–site combination. We collected distance data for all ungulates visible along both sides of the road out to 500 m. When a herd or singleton was sighted, we recorded the perpendicular distance from the road to the animal(s) location when first detected using a laser rangefinder (Bushnell Arc 1000), the total number of individuals, and the GPS position of the vehicle. If the sighting was a herd of animals, distance was measured as the perpendicular distance from the road to the approximate middle of the group. Distance data were analyzed with program DISTANCE 6.0 (Thomas et al., 2010) to estimate density of animals in each season–site combination while accounting for variation in detectability according to distance from the road transect. We analyzed distance data following recommendations in Buckland, et al., (2005). We considered all roads surveyed within a site during a single sampling event as a single transect, with two replicate samples in every season of every year, for a total of six replicates in each season–site combination. Transect lengths in km were as follows TNP = 357, MRC = 80, LMNP = 38, LGCA = 100, and MGCA = 53. We discarded the farthest 15% of observations. We plotted frequency histograms of perpendicular distances and fitted models to the histogram based on the key function and series expansion approach. We fit uniform, half‐normal, and hazard‐rate key functions with cosine and simple polynomial series expansions. We fit the key function models and associated series expansions to the data and used corrected Akaike information criterion (AICc) to select the best detection function model. We assessed goodness‐of‐fit of the top model using chi‐square and Cramer–von Misses tests. We estimated season‐ and site‐specific density using the top‐ranked model for each site, which was the half‐normal key function with cosine expansion in every case. Our density estimate of primary prey during each survey was used as an index of density during the previous intersurvey interval because each survey was conducted near the end of a given precipitation season.

The covariate of lion predation pressure was computed by dividing the density of lions by the density of primary prey in each site–season combination. When primary prey was zero, we substituted a 1 to avoid infinite values.

### 2.3 Giraffe data collection

We collected giraffe demographic data during systematic road transect sampling for photographic capture–mark–recapture (PCMR). We conducted 18 daytime surveys for giraffe PCMR data over three years between January 2012 and October 2014. We sampled giraffes three times per year near the end of every precipitation season (survey dates were short rains: 15 January–15 February, long rains: 15 May–15 June, dry: 15 September–15 October) by driving a network of fixed‐route road transects in the study area. We surveyed according to a robust design sampling framework (Pollock, 1982) with three occasions per year wherein each sampling occasion was composed of two sampling events during which we surveyed all road transects in the study area (3 occasions per year × 2 events per occasion × 3 years = 18 survey events). Road density throughout the study area was high relative to giraffe home range size (~90 km^2^ mean female home range). Driving speed was maintained between 15 and 20 kph on all transects, and all survey teams included the same two dedicated observers and a driver. Each road segment was sampled only one time in a given event. We systematically shifted the order and direction in which sites and road transects were sampled similar to a Latin square design to reduce sampling biases.

During PCMR sampling events, the entire study area was surveyed and a sample of individuals were encountered and either “marked” or “recaptured” by slowly approaching and digitally photographing the animal's right side. We photographed and later identified individual giraffes using their unique and unchanging coat patterns (Foster, 1966). We attempted to photograph every giraffe encountered for individual identification from a distance of approximately 100 m (mean = 90, *SD* = 39) and recorded sex (male, female), GPS location, and age class. We categorized giraffes into four age classes: newborn calf (0–3 months old), older calf (4–11 months old), subadult (1–3 years old for females, 1–6 for males), or adult (>3 years for females, >6 years for males) using a suite of physical characteristics, including body shape, relative length of the neck and legs, ossicone characteristics, and height (Lee, 2015; Lee, Bond, Kissui, Kiwango, & Bolger, 2016; Strauss, Kilewo, Rentsch, & Packer, 2015). For this analysis, we only utilized data from adult females and newborn calf‐age animals born during the 4‐month interval before each survey.

We matched giraffe identification images using Wild‐ID, a computer program that matched a large test dataset of giraffe images collected using our protocols with a low false rejection rate (0.007) and zero false acceptance rate (Bolger, Morrison, Vance, Lee, & Farid, 2012). Based on matching results, we created individual encounter histories for all animals for analysis. We assigned an individual to one of the five sites for the entire study according to where the majority of encounters occurred (<5% of individuals moved among sites). When no majority was present, we assigned the animal to the first observed location.

### 2.4 Estimating calf survival and neonatal survival

We used PCMR data to directly estimate calf survival during the 4‐month interval after every survey in every season–site combination (see Section 2.5 below for details). We estimated neonatal survival from variation in apparent reproduction rates (calves per adult female) during the interval before each survey in every season–site combination. These two demographic metrics, neonatal survival before each survey, and calf survival after each survey provide information about lion predation on giraffe calves during two different periods: the very vulnerable neonatal period during the first few months of life before our seasonal surveys, as well as their first season of life after they have been observed for the first time.

We computed a site‐ and season‐specific apparent reproductive rate as calves per adult female, using logistic models with the number of observed newborn calves as the number of successes, and the site‐specific $\hat{N}$ of adult females as the number of trials. The proportion of females seen with a calf as an index of reproduction is biased unless variation in the probability of detection is accounted for, along with calf mortality between birth and the first observation of the calf during surveys (Bonenfant, Gaillard, Klein, & Hamann, 2005; Nichols, 1992). Therefore, we corrected our calf counts for detectability during each survey by dividing the count by site‐ and season‐specific capture probabilities during that survey. We also corrected calf counts for the background mortality rate between birth and our survey by dividing the detectability‐corrected count by the square root of site‐ and season‐specific survival estimates for calves estimated for the appropriate interval (Caswell, 2001). In this manner, our calf counts at each site in each survey were unbiased by imperfect detectability during surveys, and also unbiased by background mortality of calves between birth and our survey.

Because we accounted for known site–season estimates of background mortality between birth and survey, we attributed any unexplained variation in apparent reproduction rate to mortality of neonatal giraffes in addition to the background mortality rate during the period immediately postbirth, before they were available to be detected by our surveys, thus providing evidence of direct or indirect predation effects. There are no data on adult female calving rate or breeding propensity from these sites, but documented calving rates show extremely low spatiotemporal variability (CV = 0.07) relative to reproductive success (CV = 0.39; Lee & Strauss, 2016). Thus, we assumed calving rate was relatively constant and variation in reproductive success was primarily due to neonatal mortality from predation. It is unlikely that lion predation pressure could directly affect birth timing of giraffes, as previous work has shown that giraffes are generally asynchronous breeders, with a possible small birth pulse that is mediated by rainfall‐triggered primary productivity affecting estrus timing (Dagg, 2014; Dagg & Foster, 1976; Hall‐Martin, Skinner, & Van Dyke, 1975; Sinclair, Mduma, & Arcese, 2000).

### 2.5 Model selection

We analyzed apparent reproduction for 860 adult females and 449 individually identified calves. We analyzed apparent calf survival using encounter histories for 449 individually identified calves. Spatiotemporal models were compared with data from five sites in three seasons each year for 15 site–season samples, all repeated over 3 years. Throughout model ranking and selection procedures for apparent reproduction and survival, we ranked models using qAICc and used model qAICc weights (*W*) as a metric for the strength of evidence supporting a given model as the best description of the data (Burnham & Anderson, 2002). We tested goodness‐of‐fit in encounter history data using U‐CARE (Choquet, Lebreton, Gimenez, Reboulet, & Pradel, 2009), and we found some evidence for lack of fit (χ_62_^2^ = 97, *p *=* *.006), but we felt this was largely due to lack of age effects in the goodness‐of‐fit tested model, whereas age effects were included in our model selection and estimation. Additionally, because the computed $\hat{c}$ adjustment was < 3 ($\hat{c}\;=\;1.5$), we did not apply a variance inflation factor (Burnham & Anderson, 2002; Choquet et al., 2009). Due to model selection uncertainty, we present model‐averaged parameter values and based all inferences on these model‐averaged parameters (Burnham & Anderson, 2002). We calculated model‐averaged real estimates by averaging from all weighted models (Burnham & Anderson, 2002). We considered spatiotemporal covariates to be statistically significant if the 85% confidence interval of the beta coefficient did not include zero (Arnold, 2010).

We used R (R Core Development Team 2013) to rank models and estimate apparent reproduction rates using generalized linear models with a logistic distribution (corrected calf counts as the number of successes and number of adult females as the number of trials) for all season–site combinations. We modeled and estimated apparent survival using Pollock's (1982) robust design statistical models in program MARK 7.1 (White & Burnham, 1999). We modeled and estimated probabilities of capture (*p*), recapture (*c*), apparent survival (*S*), and temporary emigration parameters (γ*′* and γ*″*). We began with our most parameterized model, which included survival as a linear effect of calf age, varied categorically by site, season, and site × season interaction; and with site and time effects in all other parameters: {*S*(age + site × season) γ′(site × time) γ″(site × time) *p*(site + event + season) *c*(site + event + season)}. In young ungulates, survival generally increases with age (Gaillard et al., 2000) and may also differ by sex (Clutton‐Brock, Major, & Guinness, 1985). Preliminary analyses indicated that age, but not sex, was a significant factor in giraffe calf survival (Lee, 2015).

We first ranked competing models with reduced temporal complexity of detectability parameters (*p* and *c*) and temporary emigration parameters (γ′ and γ″). Once the most parsimonious forms of detectability and temporary emigration parameters were obtained, we ranked our four spatiotemporal models of apparent survival.

## 3 Results

**Figure 2:**
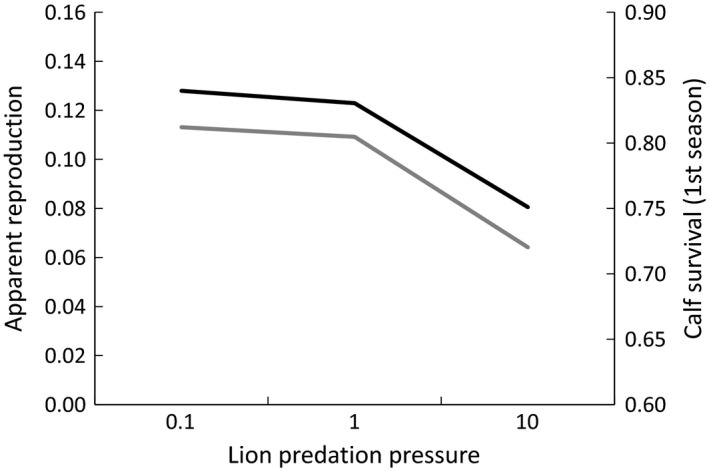
Giraffe apparent reproduction (black line; calves per adult female per year, an index of neonatal survival) and apparent calf survival (gray line; probability of survival for four months following first detection) decline with increasing lion predation pressure (lion density/primary prey density) in the Tarangire Ecosystem, Tanzania 2012–2014

We found evidence that local lion predation pressure was the best model describing spatiotemporal variation in apparent reproduction (Table 2), and calf apparent survival (Table 3). Local lion predation pressure was significantly negatively correlated with both apparent reproduction (Figure 2; β = −0.051, *SE* = 0.009, z = −5.64, *p* < .0001) and calf survival (Figure 2; β = −0.052, *SE* = 0.029, 85% CI = −0.094 to −0.011). Neither the lion density model nor the primary prey density model of spatiotemporal variation in giraffe demographic traits was statistically significant. The two significant correlations indicate that the seasonal local presence of large migratory herds of primary prey significantly reduced local lion predation pressure on giraffe neonates and calves (Figure 1) and the negative correlations both support the apparent mutualism hypothesis.

**Table 2:** Selection results for spatiotemporal covariate models of neonatal survival via apparent reproduction of giraffes

Model #	Apparent reproduction	ΔqAICc	*W*	*k*
1	Lion predation pressure	0	1.0	2
2	Primary prey density	26.56	0	2
3	Lion Density	28.26	0	2
4	Constant	31.59	0	1

Data are from the Tarangire Ecosystem, Tanzania 2012–2014. *Primary prey density* modeled apparent reproduction in each site–season combination as a function of the local density of wildebeests and zebras, and *lion density* modeled apparent reproduction as a function of local lion density. *Lion predation pressure* modeled apparent reproduction according to predation pressure (*lion density/primary prey density)*. C*onstant* is the null model. *Lion predation pressure* was significantly negatively correlated with apparent reproduction (β = −0.051, *SE* = 0.009, *z* = −5.64, *p* < .0001).

**Table 3:** Selection results for spatiotemporal covariate models of apparent local calf survival of giraffes between first detection and 4 months later

Model #	Calf survival	ΔqAICc	*W*	*k*
1	Lion predation pressure	0	0.44	95
2	Constant	0.63	0.32	94
3	Lion density	2.85	0.11	95
4	Primary prey density	2.23	0.08	95

Data are from 449 calves in the Tarangire Ecosystem, Tanzania 2012–2014. *Primary prey density* modeled calf survival in each site–season combination as a function of the local density of wildebeests and zebras, and *lion density* modeled survival as a function of local lion density. *Lion predation pressure* modeled survival according to predation pressure (*lion density/primary prey density)*. *Constant* is the null model. *Lion predation pressure* was significantly negatively correlated with calf survival (β = −0.052, *SE* = 0.029, 85% CI = −0.094 to −0.011). All models included the additional effect of *age* in survival and temporary emigration parameters, and *site* and *time* effects in detectability in the form {*S*(age) γ′ = γ″(age) *p *= *c*(site + sampling event + season)}.

## 4 Discussion

We found seasonal variation in lion predation pressure (the local density of lions divided by local density of their primary prey of migratory wildebeests and zebras) was negatively correlated with the survival of giraffe neonates and calves, supporting the apparent mutualism hypothesis. The survival effect we described is due to variable predation on giraffe calves during the seasonal changes in migratory herd local density. The seasonally changing availability of primary prey modulates the predation experienced by giraffe neonates and calves via the predator's behavior (Holt & Lawton, 1994). The positive survival effect on giraffe calves during periods of higher local ungulate density relative to lion density is likely due to predator swamping or prey switching (Ims, 1990).

The covariate models of local primary prey densities or lion densities alone were not the best descriptors of spatiotemporal variation in giraffe demographic traits—only when both predator and primary prey densities were combined as a measure of predation pressure did the model provide a good description of the data. Changes in lion behavioral or functional response appeared to begin reducing lion predation on giraffe calves when the ratio of lion density to primary prey density was between 1 and 5, resulting in the observed apparent short‐term mutualism (Figure 1). Empirical evidence for apparent mutualisms has been increasingly reported recently for a variety of taxa including: mammal predators on mammal and bird prey (Bêty, Gauthier, Giroux, & Korpimäki, 2001); plant seeds predated by rodents and birds (Kitzberger, Chaneton, & Caccia, 2007); avian predators on small‐mammal prey species (Carslake et al., 2011; Ims, Yoccoz, & Killengreen, 2011); insect–parasitoid systems (Tack, Gripenberg, & Roslin, 2011); mammalian predator–prey systems including livestock (Sundararaj et al., 2012); and mammal and avian predators on ground‐nesting bird and small‐mammal prey systems (Gauthier, Bêty, Giroux, & Rochefort, 2004; Iles et al., 2013).

Megaherbivore adults (e.g., elephants, rhinoceroses, and giraffes) are considered to be exempt from predator regulation (Sinclair, Mduma, & Brashares, 2003). Adult megaherbivores escape predator regulation because they are usually too large for predators to kill, and although predators kill some newborn animals, this is believed to have no effect on the population (Owen‐Smith, 1988; Sinclair et al., 2003). However, we found the local absence of migratory herds in the TE resulted in increased predation on giraffe calves such that calf survival was reduced 11% and apparent reproduction was reduced 37% compared with seasons when migratory herds were locally present. Our data indicate that natural predation does have a significant effect on giraffe calf and neonate survival, and reductions in calf survival rates such as we observed could significantly affect giraffe population dynamics.

Temporal scale of studies investigating indirect interactions is also important. Indirect interactions are frequently subdivided into short‐term vs. long‐term indirect interactions (Holt & Kotler, 1987; Holt & Lawton, 1994). Short‐term indirect interactions occur within a single generation, and are typically a result of behavioral responses of the predator (Holt & Kotler, 1987). Long‐term indirect interactions take place over multiple generations and are due to changes in densities of the predator and prey (Holt, 1977). In theory, both short‐ and long‐term effects of a shared predator can lead to either apparent competition or apparent mutualism. Bêty et al. (2001) documented annual nesting success of snow geese (*Anser caerulescens*) was associated positively with the overall abundance of brown lemmings (*Lemmus sibiricus*) and collared lemmings (*Dicrostonyx groenlandicus*), indicating apparent mutualism. However, Bêty, Gauthier, Korpimäki, and Giroux (2002) reported short‐term apparent mutualism and long‐term apparent competition effects of lemming populations on arctic‐nesting geese, and the outcome between these opposing indirect effects was described as apparent competition between rodents and terrestrial arctic‐nesting birds. Owen‐Smith and Mills (2008) suggested that lion selection for giraffes as prey was positively correlated with primary prey abundance over many years in South Africa's Kruger National Park, indicating long‐term apparent competition. Local long‐term apparent competition may or may not be present in the TE; to elucidate the long‐term relationship between primary prey abundance and predation on giraffe calves will require additional observation years across a large gradient of primary prey abundance within the ecosystem.

Migrating species escape from predator regulation and access ephemeral, high‐quality food resources not available to nonmigrants, thus allowing migratory populations to become an order of magnitude greater in number compared to residents (Sinclair et al., 2003). The migratory herds in the TE are threatened by increasing habitat fragmentation and restricted connectivity between wet‐season calving grounds and the dry‐season range along the Tarangire River (Morrison & Bolger, 2014) as well as high rates of illegal poaching (Kiffner, Peters, Stroming, & Kioko, 2015). Historically, primary prey in the TE was five times more abundant than the current numbers (Lamprey, 1964; Morrison, Link, Newmark, Foley, & Bolger, 2016), while lion density only halved over the same 20‐year period (Bauer, Packer, Funston, Henschel, & Nowell, 2016; Packer et al., 2011). Thus, in the past, the seasonal differences in density of primary prey would have been much greater than currently observed, and the overall predator–prey ratio would have been lower. Hatton et al. (2015) described the Africa‐wide relationship between predator and prey biomass and found a significant decline in the predator–prey ratio as prey biomass increased. This general relationship provides a prediction for the dynamics of the TE: as primary prey abundance has declined in the TE, predation pressure on giraffes has likely increased. Theoretical studies have found that when two prey species share a generalist predator such as lions, increasing fragmentation of habitat for primary prey or the extirpation of primary prey species can result in increased predation on the second prey, leading to increased secondary prey extinction rates (Abrams et al., 1998; Ryall & Fahrig, 2006). Therefore, it is conceivable that if wildebeest and zebra populations are further reduced as a result of habitat fragmentation, disrupted migration, and poaching, then giraffe calves might face increased predation pressure from local lions and other predators due to increased predator–prey ratios. Giraffe populations in the TE also are declining (Lee et al., 2016), and the additional stressor of increased natural predation following the loss of most migratory ungulate biomass could exacerbate the giraffe's population decline. Our results suggest that the widespread population declines observed in many migratory systems are likely to trigger demographic impacts in other species due to indirect effects like those shown here.

## Conflict of interest

None declared.
